# Supplementary material for: A Visible-Light-Active CuS/MoS2/Bi2WO6 Aptamer Sensitively Detects the Non-Steroidal Anti-Inflammatory Drug Diclofenac
Source: Nanomaterials (Basel). 2022 Aug 18;12(16):2834. doi: 10.3390/nano12162834 (PMC9414549; doi:10.3390/nano12162834)
Supplement: Supplementary file 1 [file nanomaterials-12-02834-s001.zip › nanomaterials-1813290-supplementary.pdf]

Supplementary Materials

A Visible-Light-Active $CuS/MoS_2/Bi_2WO_6$ Aptamer Sensitively Detects the Non-Steroidal Anti-Inflammatory Drug Diclofenac

Yun He [1], Hongjie Gao [2] and Jiankang Liu [1,*]

The Key Laboratory of Biomedical Information Engineering of Ministry of Education, School of Life Science and Technology, Xi'an Jiaotong University, Xi'an 710049, China

* Correspondence: liujiankang2021@hotmail.com

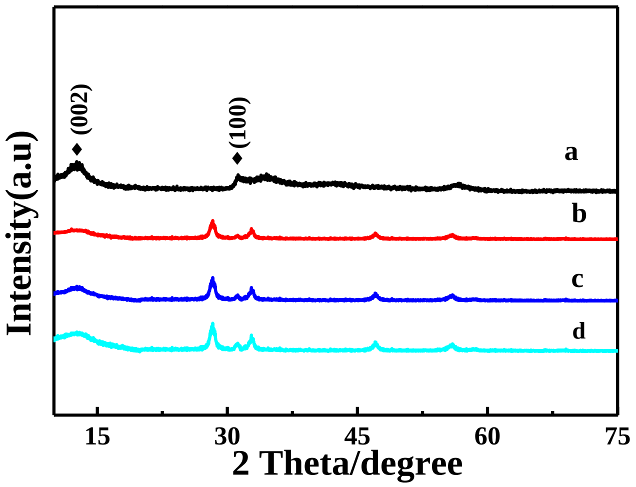

Figure S1. XRD patterns of MoS_2, MoS_2/Bi_2WO_6-2% and MoS_2/Bi_2WO_6-5%, MoS_2/Bi_2WO_6-7% nanocomposites.

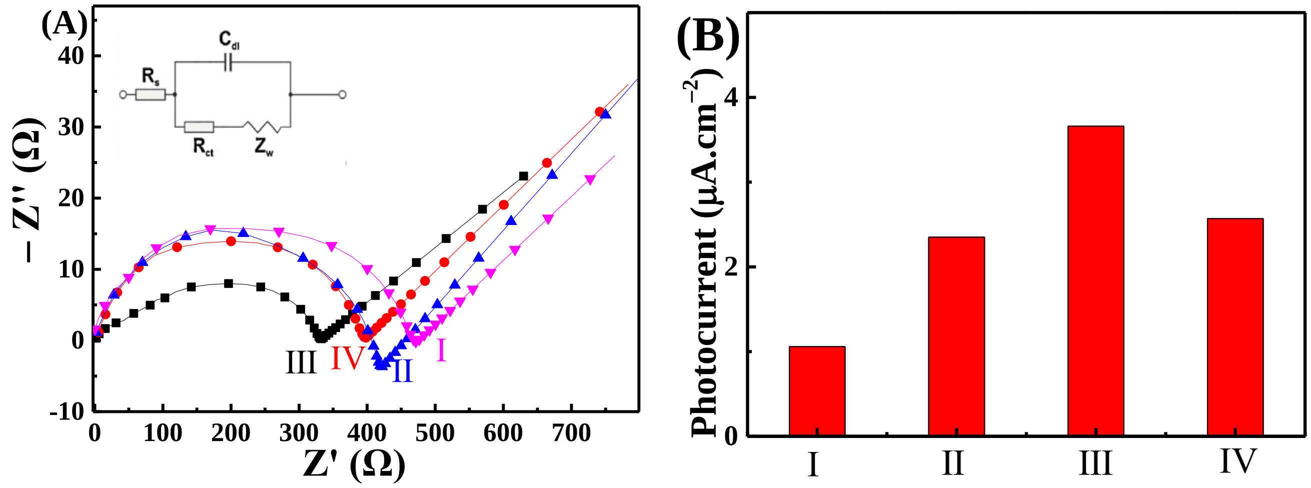

Figure S2. Nyquist plots (A) and PEC responses (B) of difffferent electrodes: (I) Bi_2WO_6, (II) MoS_2/Bi_2WO_6-2%, (III) MoS_2/Bi_2WO_6-5% and (IV) MoS_2/Bi_2WO_6-7%. The EIS spectra were achieved in 0.1 M KCl containing 5 mM Fe $(CN)_6^{3-/4-}$.

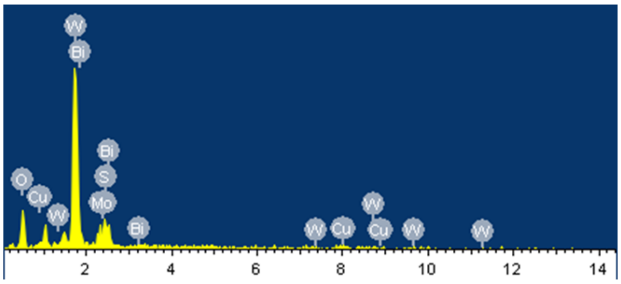

Element	O K	S K	Cu L	Mo L	W M	Bi M	Total
Weight percentage	16.69	7.83	5.21	10.43	33.23	26.61	100.00

Figure S3. EDS spectrum of $CuS/MoS_2/Bi_2WO_6$-5% microstructures.

Figure S4 shows the FTIR spectra of the as-prepared samples. Specifically, the adsorption band at 442.3 cm^{-1} is owing to the bending vibrations of Bi–O bond. A vibration peak at 596.5 cm^{-1} signifies the presence of Cu–S bond. the strong peaks at 778.1 cm^{-1} and 823.8 cm^{-1} are attributed to the W–O and W–O–W bond of Bi_2WO_6, respectively. The bands at 1380 and 3430 cm^{-1} are induced by the bending and stretching vibrations of the water molecules adsorbed on the sample surface. The results show that there is no residue of other organic matter

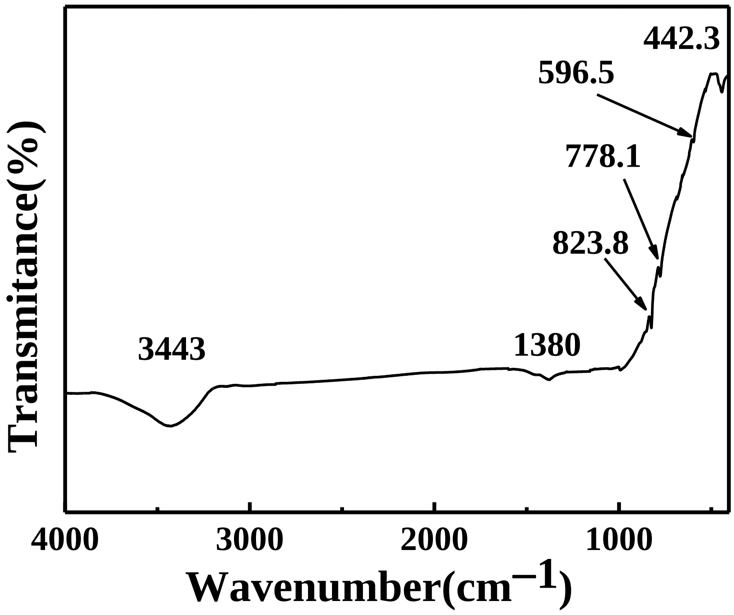

Figure S4. FT-IR spectra of the $CuS/MoS_2/Bi_2WO_6$-5% composites.

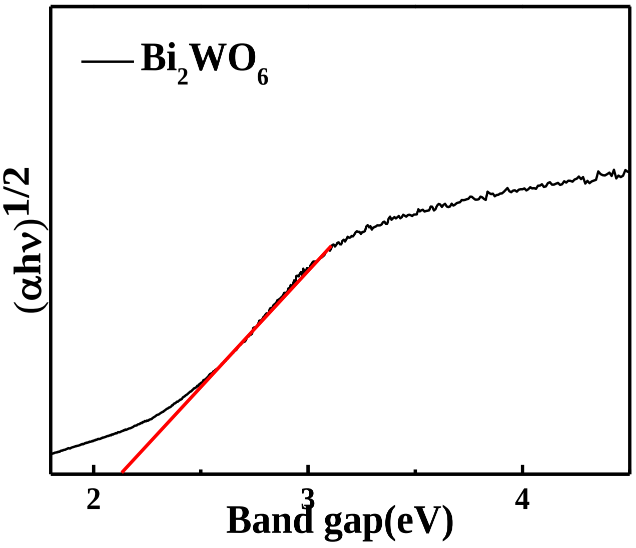

Figure S5. Plots of (αhν) n/2 vs. photon energy (hν) for Bi_2WO_6.

Table S1. Comparison of analytical performances of the $CuS/MoS_2/Bi_2WO_6$ electrodes with the typical reported methods.

Typical methods for DCF detection	Linear Range	LOD	Ref.
Photoelectrochemical aptasensor	1.0×10^{-10}–5.0×10^{-7}	6.9×10^{-11}	[1]
Photoelectrochemical aptasensor	1.0×10^{-9}–1.5×10^{-7}	7.8×10^{-10}	[2]
Impedimetric aptasensor	1.0×10^{-8}–2.0×10^{-7}	2.7×10^{-9}	[3]
Electrochemical aptasensor	0–5.0×10^{-6}	2.7×10^{-7}	[4]
HF-LPME/HPLC-DAD	5.9×10^{-10}–3.4×10^{-8}	1.8×10^{-10}	[5]
Photoelectrochemical aptasensor	1.0×10^{-10}–5.0×10^{-7}	3.3×10^{-11}	[6]
$CuS/MoS_2/Bi_2WO_6$	1.0×10^{-10}–5.0×10^{-7}	3.0×10^{-11}	This Work

References

1. Yang, L.W.; Li, L.L.; F.; Li, Zheng, H.J.; Li, T.T.; Liu, X.Q.; Zhu, J.C.; Zhou, Y.M.; Alwarappan, S. Ultrasensitive photoelectrochemical aptasensor for diclofenac sodium based on surface-modified TiO_2-$FeVO_4$ composite. *Anal. Bioanal. Chem.* **2021**, *413*, 193–203. https://doi.org/10.1007/s00216-020-02991-0.
2. Okoth, O.K.; Yan, K.; Feng, J.; Zhang, J. Label-free photoelectrochemical aptasensing of diclofenac based on gold nanoparticles and graphene doped CdS. *Sens. Actuators B Chem.* **2018**, *256*, 334–341.
3. Derikvand, H.; Roushani, M.; Abbasi, A.R.; Derikvand, Z.; Azadbakht, A. Design of folding-based impedimetric aptasensor for determination of the nonsteroidal anti-inflammatory drug. *Anal. Biochem.* **2016**, *513*, 77–86.
4. Kashefi-Kheyrabadi, L.; Mehrgardi, M.A. Design and construction of a label free aptasensor for electrochemical detection of sodium diclofenac. *Biosens. Bioelectron.* **2012**, *33*, 184–189.
5. Payan, M.R.; Lopez, M.A.B.; Fernandez-Torres, R.; Bernal, J.L.P.; Mochon, M.C. HPLC determination of ibuprofen, diclofenac and salicylic acid using hollow fiber-based liquid phase microextraction (HF-LPME). *Anal. Chim. Acta* **2009**, *653*, 184–190.
6. Shi, T.Y.; Wen, Z.R.; Ding, L.J.; Liu, Q.; Guo, Y.S.; Ding, C.F.; Wang, K. Visible/near-infrared light response VOPc/carbon nitride nanocomposites: VOPc sensitizing carbon nitride to improve photo-to-current conversion efficiency for fabricating photoelectrochemical diclofenac aptasensor. *Sens. Actuators B Chem.* **2019**, *299*, 126834.
